# Reproductive biology and variation of nuclear ribosomal ITS and ETS sequences in the *Calligonum mongolicum* complex (Polygonaceae)

**DOI:** 10.3897/phytokeys.76.10428

**Published:** 2017-01-16

**Authors:** Wei Shi, Jun Wen, Yanfeng Zhao, Gabriel Johnson, Borong Pan

**Affiliations:** 1 Key Laboratory of Biogeography and Bioresource in Arid Land, Xinjiang Institute of Ecology and Geography, Chinese Academy of Sciences, Urumqi 830011, China; 2 Turpan Eremophytes Botanic Garden, Chinese Academy of Sciences, Turpan 838008, China; 3 Department of Botany, Smithsonian Institution, PO Box 37012, Washington, DC 20013-7012, USA

**Keywords:** Calligonum mongolicum complex, Phenology, Breeding System, Crossing experiments, Phylogeny, ETS, ITS

## Abstract

To explore the biosystematics of the *Calligonum mongolicum* complex (Polygonaceae), the flowering phenological period, breeding and pollination characters and seed set of the complex (*Calligonum Mongolicum* Turze, *Calligonum chinense* A. Los., *Calligonum gobicum* A. Los., *Calligonum pumilum* A. Los. and *Calligonum zaidamense* A. Los.) were documented in the Turpan Eremophyte Botanical Garden, China. The sequences of the nuclear ribosomal ITS and ETS region were employed to differentiate the *Calligonum mongolicum* complex and other species in sect. Medusae. The results showed species of the *Calligonum mongolicum* complex occupied overlapping flowering periods and had consistent pollination agents. Their breeding systems are all self-compatible, tend to be out-crossing and they interbreed amongst each other (out-crossing index, OCI = 4).The crosses within and amongst species had high seed sets (44 - 65%). Phylogenetic analyses of Calligonum sect. Medusae and the network analysis of nrDNA (ITS and ETS) in the complex suggest interbreeding amongst “species” within the complex and provide evidence for taxonomically merging the five species in the complex. The detected hybridisation, occurring within the complex, suggests the need to improve traditional methods of *ex situ* plant conservation in botanical gardens for maintaining genetic diversity of *Calligonum* within and amongst species from different geographic areas.

## Introduction

*Calligonum* L. is widely distributed in Northern Africa, Southern Europe and Western and Central Asia (Bao and Alisa 2003). It is the only genus in Polygonaceae that contains C_4_ species (Pyankovet al. 2000) with rapid rates of evolution and diversification (Mabberley 2008). The taxonomy of this genus is complex (Xu 1998) and that of the *Calligonum mongolicum* Turcz. complex is especially difficult. *Calligonum mongolicum* Turcz. is widely distributed from Xilinhot-Inner Mongolia in the east, Kyzyl Kum Desert in Uzbekistan in the west, Milan in Xinjiang in the south, Baitashan, Qitai and Karamay in Xinjiang in the north, with a longitudinal range of about 30° (Pavlov 1936; Drobov 1953; Baitenov and Pavlov 1960; Sergievskaya 1961; Kovalevskaja 1971; Shi et al. 2011). *Calligonum pumilum* A. Los., *Calligonum gobicum* A. Los., *Calligonum chinense* A. Los., *Calligonum alashanicum* A. Los., *Calligonum zaidamense* A. Los. and *Calligonum roborowskii* A. Los. (1927) of the complex occur within the geographic range of *Calligonum mongolicum* (Losinskaja 1927; Bao and Grabovskaya-Borodina 2003). All of these more narrowly ranged species were merged into *Calligonum mongolicum* based on the variation of their fruit characters and the chromosome numbers (Soskov 1975a, 1975b). However, these species are currently recognised in the Flora of China treatment according to their fruit morphology (Bao and Grabovskaya-Borodina 2003; Mao et al. 1983). Nevertheless the fruits are overall similar, making it difficult to distinguish the species of the complex (Soskov 2011, Mao and Pan 1986, Shi et al. 2011; Table 1). Analyses of the reproductive biology of the complex are important for resolving the taxonomy and exploring the evolutionary processes (Stebbins 1950; Grant 1992, 1994; Oldfield 2009).

**Table 1.** Differences in fruit characters among species of the *Calligonum mongolicum* complex according to the treatment in Flora of China, the monograph of Soskov (2011) and the observations by Shi et al. (2011). * NRR = Number of rows of bristles in each rib.

Fruit morphology	*Calligonum mongolicum*			*Calligonum pumilum* (syn. *Calligonum rubescens*)			*Calligonum chinense* (syn. *Calligonum litwinowii* Drob.)			*Calligonum gobicum* (syn. *Calligonum litwinowii* Drob.)			*Calligonum zaidamense* (syn. *Calligonum litwinowii* Drob.)		
	Flora of China	Soskov (2011)	Shi et al. (2011)	Flora of China	Soskov (2011)	Shi et al. (2011)	Flora of China	Soskov (2011)	Shi et al. (2011)	Flora of China	Soskov (2011)	Shi et al. (2011)	Flora of China	Soskov (2011)	Shi et al. (2011)
Fruit length (mm)	8–12	8–12	5–15	7–12	12–22 mm	5–17	10–15	9–12	8–13	11–18	9–12	10–12	10–17	9–12	11–18
Seta length (mm)	–	3.5–5	1–5	–	(3)5–8(10) mm	1–5	–	3.5–5	2–7	–	3.5–5	2–4	–	3.5–5	3–6
NRR*	2 or 3	(1)2(3)	2 or 3	1	(2)3	1 or 2	3	2 or 3	2 or 3	2	2 or 3	2	2	2 or 3	2
Ribs flat or elevated	prominent or not	flat	prominent or not	–	elevated	prominent or not	flat	little elevated	flat	flat	little elevated	flat	flat	little elevated	flat
Seta texture & branching	soft, thin, 2 or 3 -branched	soft, thin, 2-branched	soft, thin, 2, 3 or 4 branched	soft, thin, 2 or 3-branched	soft, 3–4 -branched	soft, thin, 2, 3 or 4 branched	thick, stiff, 2 or 3 branched	thick, 3 or 4 branched	thick, stiff	thick, breakable, 2-branched	thick, 3 or 4 branched	thick, breakable	thick, breakable, 2-branched	thick, 3 or 4 branched	thick, breakable
Seta distance (mm)	–	moderately dense	0.2–2	–	0.7–1	1–2	–	0.5–1.2	0.5–2	–	0.5–1.2	0.1–1.8	–	0.5–1.2	1.2–2.3
Nutlet length (mm)	–	9–10	5–10	–	7–10	5–12	8–11	6–10	1.5–9.2	–	6–10	6.7–8.2	–	6–10	7.2–12
Nutlet width (mm)	–	2.8–3	2–6	–	3–3.5	2–5	3–5	4–5	3.6–9.8	–	4–5	3.0–4.1	–	4–5	3.1–7.2
Nutlet coiled or not and its form	not coiled, ellipsoid	not coiled	coiled or not	coiled, ovoid	coiled	coiled or not	coiled, ellipsoid	coiled	coiled, ellipsoid	not coiled, oblong	coiled	not coiled, ellipsoid	not coiled, broadly ovoid or ellipsoid	coiled	not coiled, broadly ovoid or ellipsoid

Studies on the reproductive biology of *Calligonum* are rare. Kang et al. (2011) assessed information from four taxa (*Calligonum calliphysa* Bunge, *Calligonum rubicundum* Bge., *Calligonum densum* Borszcz and *Calligonum ebinuricum* Ivanova) which were selected from each section (four sections in *Calligonum*) and revealed that all the investigated species were self-compatible but there was no hybridisation amongst them. A few examples of hybridisation were mentioned such as between *Calligonum dubjanskyi* Litv. and *Calligonum bubuyri* B. Fedtsch. ex Pavl., between *Calligonum acanthopterum* and *Calligonum leucocladum* and between *Calligonum acanthopterum* Borszcz. and *Calligonum leucocladum* (Schrenk) Bunge (Soskov 1975b). These reported hybrids occurred between species within a section, including sect. *Peterococus* and sect. *Medusae*. The taxonomic relationships of the genus have been tested by the applications of several molecular techniques, such as the RAPD markers (Ren et al. 2002) and other chloroplast DNA markers (*trn*L-F, *mat*K, *atp*B-*rbc*L, *psb*A-*trn*H, *psb*K-*psb*l and *rbc*L) (Tavakkoli et al. 2010; Sanchez et al. 2011; Abdurahman and Sabirhazi 2012; Sun and Zhang 2012; Li et al. 2014), but the markers employed so far have been inefficient for resolving the taxonomic problems in *Calligonum*. It was expected that reproductive biology and faster-evolving nuclear DNA sequences (Sang 2002; Zimmer and Wen 2012) might shed some light on the taxonomy of the genus.

The *Calligonum mongolicum* complex is almost exclusively diploids with 2*n* (2*x*) = 18, except *Calligonum roborowskii* with 2*n* (4*x*) = 36 (Wen et al. 2016), although a polyploid count was reported as 2*n* (3*x*) = 27 (Shi et al. 2013) in an individual of *Calligonum mongolicum*. The situation is markedly different in other species of the *Calligonum* sect. *Medusae* which are polyploids with the most frequent chromosome number 2*n* (4*x* or 6*x*) = 36 or 54 (Wang and Yang 1985; Wang and Guan 1986; Shi and Pan 2015). The above chromosomal data indicate the significant role of polyploidy in the evolution of the sect. *Medusae* of *Calligonum*. The flowering phenology, characters of breeding systems and pollination and fruit set of the *Calligonum mongolicum* complex (*Calligonum mongolicum*, *Calligonum pumilum*, *Calligonum chinense*, *Calligonum alashanicum* and *Calligonum zaidamense*) have been documented by the authors, leaving out the tetraploid *Calligonum roborowskii* (see also Wen et al. 2016). The phylogeny of *Calligonum* sect. *Medusae* has been reconstructed using nuclear ribosomal markers (ITS and ETS). The new data will be used to discuss the taxonomic implications of the species complex and the conservation strategy of *Calligonum* in botanical gardens.

## Materials and methods

Five species of the *Calligonum mongolicum* complex (*Calligonum mongolicum*, *Calligonum pumilum*, *Calligonum chinense*, *Calligonum alashanicum* and *Calligonum zaidamense*) were selected by the authors, leaving out the tetraploid *Calligonum roborowskii*. These selected species were brought to Turpan Eremophytes Botanical Garden (TEBG) from their natural habitats during 2011 to 2013 and were planted in the germplasm garden of *Calligonum* (Table 2, Qi and Pan 2010; Shi et al. 2013).

**Table 2.** Voucher information for the samples used in the study.

Species	Pop.	individuals (flowers in an individual)	Location	Num. in DNA analysis	Coordinates	
					ITS	ETS
*Calligonum mongolicum*	M1	3(25)	Erlianhaote, Neimeng, China E112°03' N43°45' 898 m	M1–2	KU050839	KY316968
				M1–3	KU050840	KY316961
	M2	3(25)	Qingtongxia, Ninxia, China E105°55' N38°01' 1134 m	M2–1	KU050847	KY316966
				M2–2	KU050853	KY316970
	M3	3(25)	Erjinaqi, Inner Mongolia China E100°26' N41°27' 1002 m	M3–1	KU050846	KY316971
				M3–2	KU050848	KY316973
				M3–3	KU050838	KY316979
	M4	3(30)	Wuerhe, Kelamayi, Xinjiang, China E 85°45’ N 46° 9’ 521 m	M4–1	KU050849	KY316969
				M4–3	KU050850	KY316972
*Calligonum pumilum*	P1	3(50)	Hami, Xinjiang, China E091°32 N43°23' 1038 m	P1–1	KU050851	KY316974
				P1–2	KU050852	*
				P1–3	KU050841	KY316960
	P2	3(25)	Hami, Xinjiang, China E091°23' N43°20' 1273 m	P2–3	KU050843	KY316962
	P3	3(25)	Liuyuan, Gansu, China E095°28' N95°28' 1744 m	P3–1	KU050844	KY316963
				P3–2	KU050845	KY316975
*Calligonum chinense*	C1	3(100)	Zhangye, Gansu, China E100°18' N39°28' 1458 m	C1–2	KY316981	KY316977
*Calligonum gobicum*	G1	3(100)	Mingqing, Gansu, China E102°52' N38°34' 1369 m	–	–	–
*Calligonum alashanicum*	A1	3(100)	Erjinaqi, Inner Mongolia China E100°27' N41°43' 969.8 m	A1–2	KY316980	KY316967
*Calligonum zaidamense*	Z1	3(100)	Zhangye, Gansu, China E100°18' N39°03' 1458 m	Z1–1	KY316982	KY316978
				Z1–2	KY316983	KY316965
*Calligonum calliphysa*		1		*Calligonum calliphysa*	KX186585	KY316976
*Calligonum arich*		6			KC585438	–
					KC585446	–
					KC585445	–
					KC585444	–
					KC585477	–
					AB542775	–
*Calligonum comosum*		2		*Calligonum comosum*	KC585417	–
					KC585430	–
*Calligonum caput-medusae*		1			JB187106	–
*Calligonum ebinuricum*		1		*Calligonum ebinuricum*	JQ731664	–
*Calligonum ebinuricum*		1		*Calligonum ebinuricum*	JQ731665	–
*Calligonum ebinuricum*		1		*Calligonum ebinuricum*	JQ731663	–
*Calligonum molle*		1			GQ206245	–
*Calligonum crinitum*		1			AB542776	–
*Calligonum junceum*		1		*Calligonum junceum*	GQ206243	–
*Calligonum junceum*		1		*Calligonum junceum*	AB542774	–
*Calligonum junceum*		1		*Calligonum junceum*	JX987230	–
*Calligonum polygonoides*		1			AB542776	–
*Calligonum mongolicum*		1		*Calligonum mongolicum*	JX259384	–
*Calligonum mongolicum*		1		*Calligonum mongolicum*	JX259385	–
*Calligonum roborowskii*		1		*Calligonum roborowskii*	JX259386	–
*Calligonum roborowskii*		1		*Calligonum roborowskii*	JX259387	–
*Calligonum takemakanense*		1		*Calligonum takemakanense*	JX259390	–
*Calligonum persicum*		1		*Calligonum persicum*	AB542777	–

### Collection of phenological information

Phenological information of the *Calligonum* species was collected from field investigations. The phenological observations were made once every two days during the growing period, according to the method of the Chinese Phenological Observation Standard (Zhu and Wan 1973). The investigated flowering phenological periods included flower bud appearance, beginning of flowering, flower blooming, end of flowering and fruit maturity. The starting date of a species’ growing period was expressed in the day of year (calculated from 1 January of the current year and thereafter).

Five plants from each species in the field were randomly selected to document the flowering phenology and they were observed every day in the blooming and fruiting periods from 2011 to 2013.

### Pollen morphology


 Scanning electron microscopy (SEM) was used to document the micromorphology of pollen. Samples were dehydrated and were then placed on aluminium stubs using double-sided adhesive tape and sputter coated with gold in a Hitachi E-1010 Ion Sputter Coater, following Wen and Nowicke (1999). The materials were subsequently observed and photographed under a Hitachi S-4800 scanning electron microscope. Pollen sizes from both polar view (P) and equatorial view (E) were measured using 10 grains of each sample.

### Controlled crossing experiments and observations on fruit and seed sets

The breeding systems of the *Calligonum mongolicum* complex were examined by a hand-pollination test. More than 1600 buds were marked and bagged before opening during the period 2011 to 2013. Each flower of an individual plant was randomly assigned to one of the following treatments with each treatment, except hybridisation, including about 30 flowers in each taxon: i) autonomous pollination: no treatment but just bagging to test self-pollination naturally; ii) selfing: test for self-compatibility by bagging and undertaking pollination from the same flower; iii) geitonogamous selfing: emasculation, bagging and pollination in the same individual but using different flowers, to test for self-compatibility; iv) crossing: emasculation, bagging and pollination from another individual that was located more than 2m from the recipient v) apomixis: emasculation, bagging but no pollen; vi) natural pollination: emasculation, no bagging; vii) autonomous pollination via geitonogamy: bagging the whole branch; viii) hybridisation: emasculation and cross-pollinations with four other species, each species included 100 flowers. The stigma receptivity time was about 12 hours; and the pollen viability was about 12-24 hours (XS Kang, W Shi and BR Pan, unpublished data).

### DNA extraction, amplification and sequencing

Nineteen (19) individuals of six species, *Calligonum mongolicum*, *Calligonum pumilum*, *Calligonum chinense*, *Calligonum alashanicum*, *Calligonum zaidamense* and *Calligonum calliphysa* were sequenced and 24 ITS sequences of *Calligonum* from GenBank were downloaded (Table 2). Young green branches of each species were collected from natural populations in China (Table 2). The samples were collected from adult individuals with green healthy branches (with no signs of parasitism or of drought stress). They were dried in silica gel and kept in a freezer at -25 °C. Voucher specimens of the studied material were deposited in the Herbarium of Institute of Ecology and Geography in Xinjiang (XJBI).

Total genomic DNAs were extracted from fresh or silica gel dried assimilating branches following the protocol of Doyle and Doyle (1990). In this study, the protocols were followed for obtaining ITS sequences in plants by Wen and Zimmer (1996), Stanford et al. (2000) and Feliner and Rossello (2007). The ETS primers were newly designed for the study with the forward primer ETScalli1: 5’-GTTACTTACACTCCCCACAACCCC-3’ and the reverse primer as18SIGS: 5’-GAGACAAGCATATGACTACTGGCAGGATCAACCAG-3’. The DNA amplifications via a polymerase chain reaction (PCR) were performed using 10 ng of genomic DNA, 4 pmol of each primer, 0.5 U Taq polymerase (Bioline, Randolph, MA, USA) and 2.5 mM MgCl_2_ in a volume of 25 µL using a PTC-225 Peltier thermal cycler. The PCR cycling parameters were as follows: 95 °C initial heating for 5 min, 40 cycles of 94 °C for 30s, 55°C for 45s for ITS (60°C for 40s for ETS) and 72°C for 60s and 72 °C for 10 min for final extension. The PCR products were purified using EXO-SapIT (US Biological, Swampscott, MA, USA) and sequenced in both directions using PCR primers. The ABI Prism Big Dye Terminator Cycle Sequencing Ready Reaction kit (Applied Biosystems, Foster City, CA, USA) was carried out for cycle sequencing with mixing in a 10 µL reaction volume including 5 ng of primer, 1.5 µL of sequencing dilution buffer and 1 µL of cycle sequencing. The conditions were as follows: 35 cycles of 96 °C for 30s denaturation, 50 °C for 30s annealing and 60 °C for 4 min elongation. An ABI 3730xl DNA analyser (Applied Biosystems, Foster City, CA, USA) was used for separating the sequencing products. Both strands of DNA with overlapping regions ensured that each base was double-checked. We assembled the electropherograms and generated the consensus with Sequencher 4.5 (GeneCodes, Ann Arbor, MI, USA).

Sequences were initially aligned using MUSCLE 3.8.31 (Edgar 2004), followed by manual adjustments using GENEIOUS 8.1.2 (Kearse et al. 2012). The newly generated sequences from the 20 samples of *Calligonum* were deposited in GenBank (Table 2). The jModeltest 2.1.7 (Posada 2008, Darriba et al. 2012) was used to show the best-fit model of sequence evolution for each data. The Bayesian inferences were run according to the model chosen by the Akaike information criterion (AIC) method. Phylogenetic relationships were inferred using both maximum-likelihood estimation (ML) in RAxML (Stamatakis 2006) and Bayesian inference (BI) in MrBayes 3.1.2 (Ronquist and Huelsenbeck 2003). Bayesian analyses were conducted using the combined ITS and ETS data sets, using partitions of the respective models from the jModeltest. The ML analyses also used the partition of the two markers. The bootstrap analysis (Felsenstein 1985) was executed with 1000 replicates, with a maximum of 100 trees saved per replicate. The Bayesian inference was run with 2,000,000 generations and the Markov chain Monte Carlo (MCMC) run had one cold and three incrementally heated chains. For each dataset, all Bayesian analyses produced split frequencies of less than 0.01 and convergence between the paired MCMC runs were repeated twice to avoid spurious results. The remaining trees were used to construct majority-rule consensus trees after discarding the first 2000-5000 trees as burn-in before stationary conditions were established. A neighbour-net analysis was conducted using the uncorrected p-distance between individuals and the programme SplitsTree 4.13.1 (Huson and Bryant 2006). Branch support was tested using bootstrapping with 1000 replicates.

## Results

### Phenological data

The bisexual flowers occur in groups of two to four in assimilating branches of the *Calligonum* species. The perianth has five tepals, which are green or red with a broad white margin abaxially, ovate, unequal and persistent in fruits. The flower has 12-18 stamens and the filaments are connate at the base. The pollen presentation pattern is gradual and, when pollen is viable, the stigmas also have receptivity (no dichogamy) (BR Pan, unpublished data).

**Figure 1.**
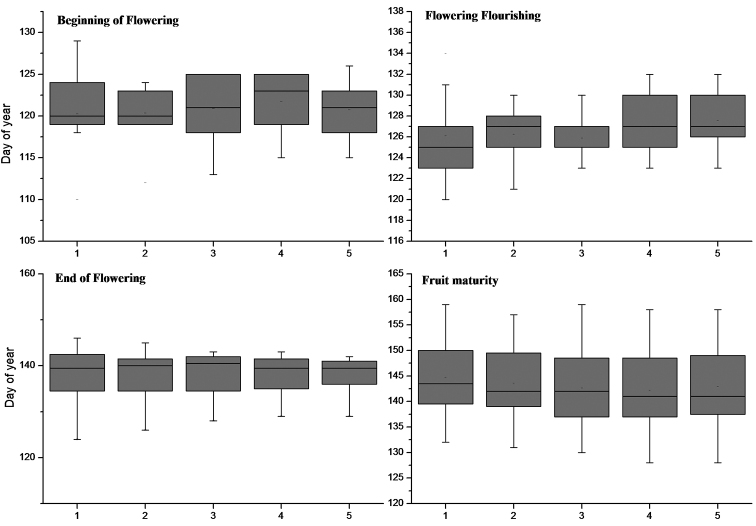
The phenological phases of the *Calligonum mongolicum* complex. **1** *Calligonum mongolicum* **2** *Calligonum chinense* **3** *Calligonum gobicum* **4** *Calligonum pumilum* and **5** *Calligonum zaidamense*.

The five *Calligonum* species flower from mid-April to mid-May in the field. The duration of *Calligonum mongolicum* and *Calligonum gobicum* for flowering was generally from mid-April to early May, whereas that of *Calligonum pumilum*, *Calligonum chinense* and *Calligonum zaidamense* was from late April to mid-May; individual species of *Calligonum mongolicum* continued to flower sporadically until late May. Thus the blooming period was similar for *Calligonum* both in field and in TEBG (Figure 1).

The blooming periods of the complex overlapped and the percentage overlap was about 80–100% (Figure 1). The peak flowering periods of *Calligonum mongolicum* complex occurred at the same time in early May. Although flowering was generally ending in early May, flowering in some individuals of *Calligonum mongolicum* was still at its peak until mid-May.

### Floral visitors

The major pollinators for collecting pollen and nectar were *Apismellifera* L. and *Halictus* sp., both of which collected pollen in pollen baskets on their third legs and, occasionally, pollen also adhered to their chests and then contacted with the stigmas whilst feeding. These species frequently visited nearby flowers on the same plant individual and frequent visits on the same flowers were also undertaken. Other recorded species were nectar thieves including some flies (*Lasiopticus* sp., *Musca domestica* and *Calliphoravicina*), butterflies (*Plebejusargus*) and others in Formicidae.

### Breeding systems

**Figure 2.**
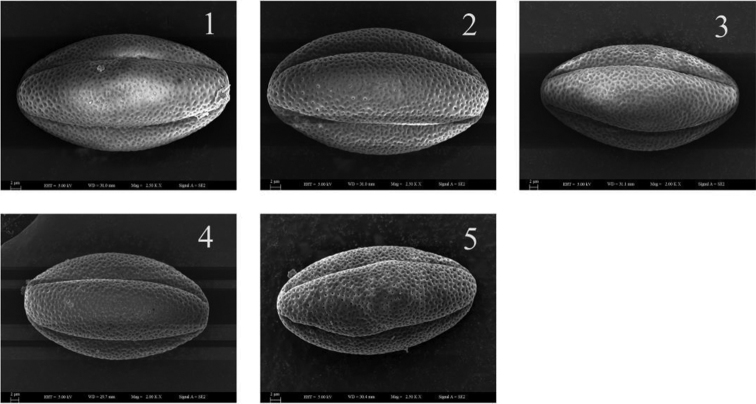
Equatorial view of pollen grains of the *Calligonum mongolicum* complex under SEM micrographs.**1** *Calligonum mongolicum* **2** *Calligonum chinense* **3** *Calligonum gobicum* **4** *Calligonum pumilum* and **5.** *Calligonum zaidamense*.

The results of the pollination experiment suggested that species in the complex had analogous mating systems (Tables 3 & 4), as both geitonogamy and cross-pollination conducted by hand yielded better fruit sets compared with natural pollination. They also had similar pollen characters and indices (P & E) (Table 5 & Figure 2). They interbred amongst each other (OCI = 4). The spontaneous self-pollination did not occur because when pollinators were excluded in the bagging treatment, no fruits were produced. It resulted in a very low (if any) fruit set in the self-pollination treatment. The fruit set using geitonogamy treatment shows self-compatibility within each species. The apomixis did not occur in these species as exclusion of both pollinators and emasculation did not result in any fruit set.

**Table 3.** Comparison of actual fruit set of species in the *Calligonum mongolicum* complex under each pollination treatment in 2011 to 2013 (n = the total number of flowers manipulated in each treatment, data shown are mean ± SE).

Treatment	Species				
	*Calligonum mongolicum*	*Calligonum gobicum*	*Calligonum chinense*	*Calligonum pumilum*	*Calligonum zaidamense*
No emasculation, bagged, self-pollination	0	0	0	0	0
Emasculation, bagged, hand geitonogamy	2.00±1.00	1.67±0.58	1.00±1.00	1.00±1.00	1.00±1.00
Emasculation, bagged, hand cross pollination in same individual	15.12±1.00	16.58±1.22	17.24±1.31	17.32±1.23	14.42±1.25
Emasculation, bagged, no pollination	0.00	0.00	0.00	0.00	0.00
Emasculation, unbagged, natural pollination	11.21±2.13	9.15±2.54	12.48±2.41	12.47±1.21	13.56±2.15
Unemasculation, unbagged, natural pollination	11.23±1.23	15.45±1.58	8.35±3.35	14.28±3.69	10.25±2.36

**Table 4.** Fruit set (%) for the five *Calligonum* species under different cross-pollination treatments (n = the total number of flowers manipulated in each treatment, mean ± SE).

Species cross	*Calligonum mongolicum* ♂	*Calligonum gobicum* ♂	*Calligonum chinense* ♂	*Calligonum pumilum* ♂	*Calligonum zaidamense* ♂
*Calligonum mongolicum* ♀	65±1.25	54±3.21	41±1.15	47±1.68	45±1.25
*Calligonum gobicum* ♀	47±2.34	44±2.47	59±4.21	57±1.51	47±2.36
*Calligonum chinense* ♀	58±1.21	46±2.11	59±4.18	66±2.12	48±3.25
*Calligonum pumilum* ♀	48±2.24	59±4.56	54±3.06	65±2.14	52±2.48
*Calligonum zaidamense* ♀	44±2.14	58±1.63	47±1.85	60±1.23	51±4.21

Hybridisation experiments in the complex resulted in a fruit set and the results (in percentage terms) are shown in Table 4. The flowering of the complex was synchronised. The pollen morphology of the five species showed similarities in major pollen characteristics such as shape, size and exine characters (Figure 2, Table 5). The hybridisation experiments and interspecific hand pollination yielded some viable seeds (Table 5). The maximum of the fruit set is amongst the *Calligonum mongolicum* (65±1.25) and the *Calligonum pumilum* (65±2.14) themselves; the minimum is that between *Calligonum chinense* and *Calligonum mongolicum* (41±1.15). In general, the fruit set amongst the five species is similar (p>0.05).

**Table 5.** The characteristics of the pollen grains of five species of the *Calligonum mongolicum* complex.

Species	Shape	Length (μm)	width(μm)	P/E	Aperture	ornamentation
*Calligonum mongolicum*	*Prolate*	38.90	23.20	1.68	tricolporate	reticulate
*Calligonum gobicum*	*Prolate*	38.35	19.51	1.97	tricolporate	reticulate
*Calligonum chinense*	*Prolate*	33.45	21.15	1.58	tricolporate	reticulate
*Calligonum pumilum*	*Prolate*	31.52	22.40	1.41	tricolporate	reticulate
*Calligonum zaidamense*	*Prolate*	37.79	20.04	1.89	tricolporate	reticulate

### Phylogenetic analysis

The aligned matrix with 45 accessions of nrITS and ETS is 807bp long. The Phi test did not find statistically significant (p= 0.0323) evidence for the presence of chimeric sequences in the nrITS and ETS data matrix. The nrITS and ETS sequence alignment used for phylogenetic tree reconstruction included 44 sequences: 43 from the in-group and one of *Calligonum caput-medusae* as the out-group. The data sets included 20 newly generated nrITS, 23 ITS sequences from GenBank and 20 new ETS sequences (Table 2).

**Figure 3.**
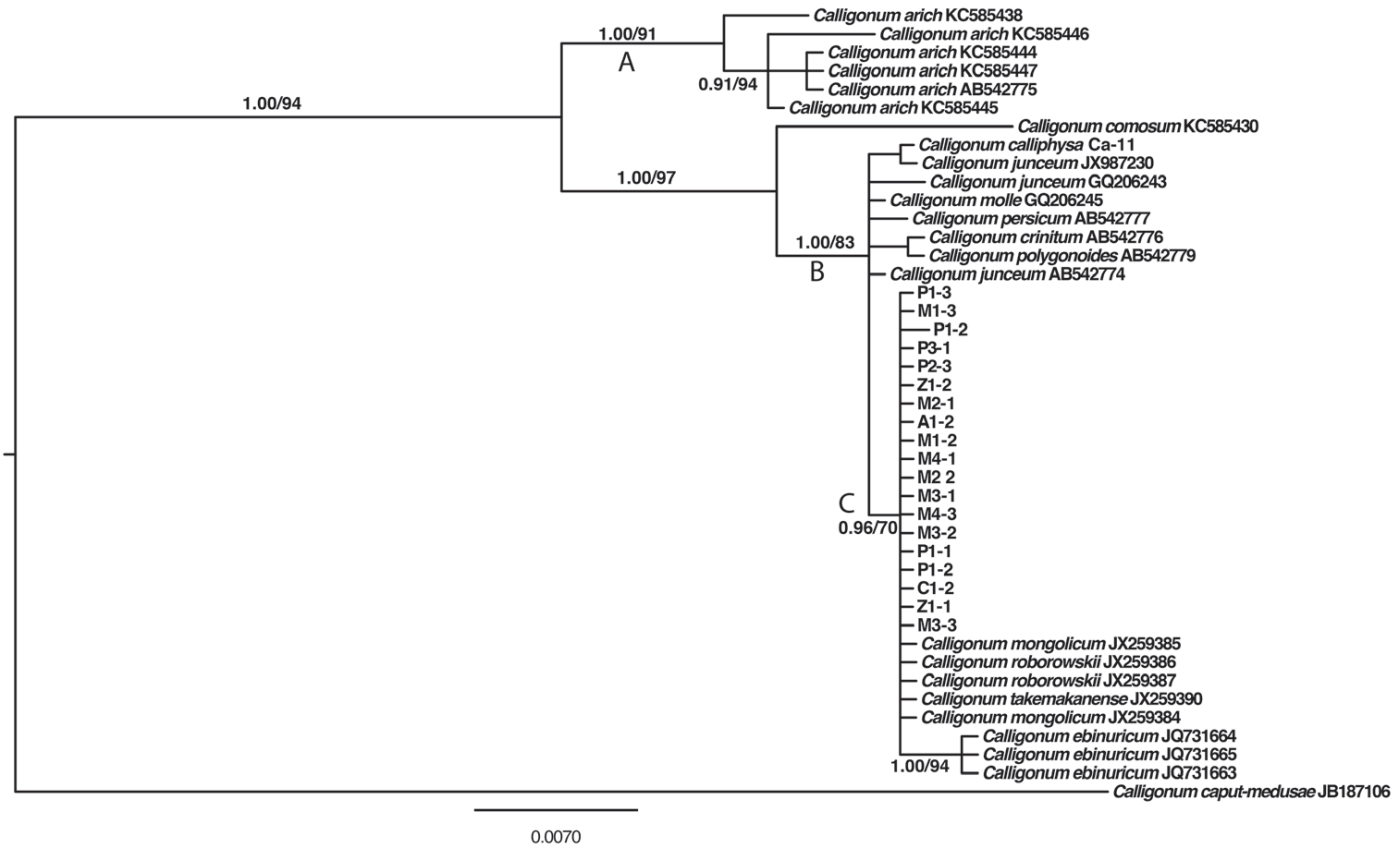
Maximum likelihood tree for 43 (in-group) *Calligonum* nrITS and ETS sequences produced with RAxML. Numbers adjacent to (relevant) nodes represent maximum likelihood value and Bayesian posterior probabilities. Branches marked with an asterisk collapse on the maximum likelihood strict consensus tree of the same dataset. The branch marked with a number sign collapses on the Bayesian majority rule consensus tree of the same dataset.

**Figure 4.**
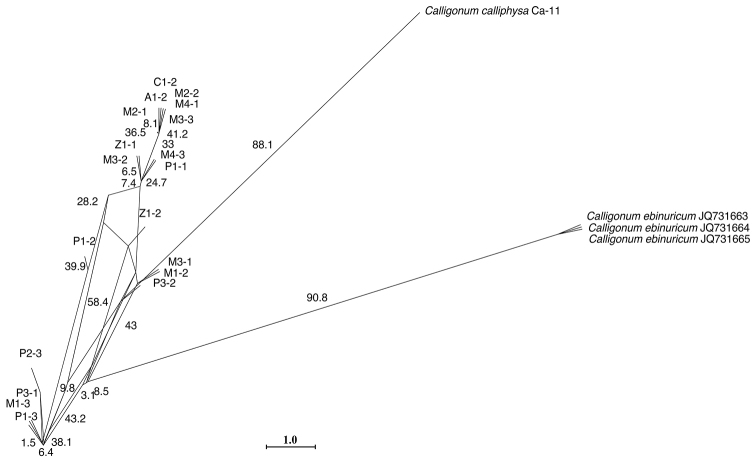
Neighbour-net analyses of the *Calligonum mongolicum* complex, *Calligonum ebinuricum*, *Calligonum calliphysa* and closely related taxa based on uncorrected p-distances. Numbers indicate bootstrap values over 1000 replicates.

The model test suggested F81 for ETS (nucleotide frequencies A = 0.2023, C = 0.3494, G = 0.2778, T = 0.1706) and TPM2uf for ITS (nucleotide frequencies A = 0.1873, C = 0.3265, G = 0.3277, T = 0.1586; substitution rates: RAC = 0.3484, RAG = 3.4478, RAT = 0.3484, RCG = 1.0000, RCT = 3.4478, RGT = 1.0000). The Bayesian inference used the partition of ITS and ETS based on the respective models. The ML analyses used GTR+G as the model. Topologies inferred by the two phylogenetic tree reconstruction methods were congruent (Figure 3). The most morphologically distinctive *Calligonum caput-medusae* from Central Asia was used as the out-group, the first diverged clade in the analyses being *Calligonum arich* (six accessions included, PP 1.00, BS 92) from western Asia, the remaining species forming a large clade A. Of interest, all species from the *Calligonum mongolicum* complex formed a clade. The five species of the *Calligonum mongolicum* complex, *Calligonum ebinuricum* and two other species *Calligonum roborowskii* and *Calligonum taklamakan* were distributed within the broad geographic region of the *Calligonum mongolicum* complex, but *Calligonum roborowskii* and *Calligonum taklamakan* were of a more restricted distribution in the Taklamakan Basin of Xinjiang province, China. The three individuals of *Calligonum ebinuricum* which form an independent clade, have specific fruit characters different from the complex. The individuals of *Calligonum mongolicum* and *Calligonum pumilum* each did not form a clade, but they were intermixed with *Calligonum alashanicum*, *Calligonum zaidamense* and *Calligonum chinense*, *Calligonum roborowskii* and *Calligonum taklamakan*, forming a large clade C (Figure 3). It is of interest to note that the p-distance amongst taxa of *Calligonum* for the ITS and ETS region is as high as 11.364% between the out-group species *Calligonum caput-medusae* and *Calligonum mongolicum* JX259384. Within the clade C, the p-distance was as high as 0.564% between *Calligonum ebinuricum* and *Calligonum mongolicum* JX259384. A neighbour-net was constructed for the *Calligonum mongolicum* complex using ITS and ETS sequences which also supported the complex in one branch (Figure 4).

## Discussion

### Evidence for interbreeding of species in the *Calligonum mongolicum* complex

Species isolation is frequently caused by the temporal heterogeneity of blooming amongst sympatric species (Levin 1971; Adams 1983; Grant 1992, 1994). The flowering periods of five species in the complex showed a high degree of overlapping, with some differences in peak blooming periods (also see cases in Wilson 1983; Burd 1995).

These five diploid species of *Calligonum* have similar pollen characters in both with spheroidal shape and tricolporate apertures with each other (Figure 2). The other species in *Medusae* also have the similar pollen characters but without specific pollen indexes (P&E) analysis (Qiu 1988; Gulinuer 2008). The hand-pollination tests suggested the five species are self-compatible (geitonogamous, not autophilous). Furthermore, pollinators were necessary for the sexual reproduction in the complex, although some fruit sets were resulted with exclusion of pollinators. The results of test crosses suggest the existence of a strong internal hybridisation potential in each of these species.

Crossing compatibility between the species of the *Calligonum mongolicum* complex is largely the same as that between individuals within the same species (Table 4). The crossing behaviour amongst them is consistent with the view from Soskov (1975a, 1975b) by treating these various segregate species as one variable biological species of *Calligonum mongolicum*.

### Lack of phylogenetic structure and nrDNA sequence variation as indirect evidence for interbreeding in the *Calligonum mongolicum* complex

Although phylogenetic inference based on nrITS needs to be considered carefully (Alvarez and Wendel 2003, Feliner and Rossello 2007), some conclusions may be drawn based on the ITS and ETS analyses of the target species. As shown by the ML and Bayesian trees of nrITS and ETS sequences (Figure 3), a striking divergence exists between *Calligonum arich* (clade A) and other species. Yet species of the *Calligonum mongolicum* complex had very similar or identical sequences (Clade C in Figure 3). The nrITS and ETS tree together with the network of ribotypes (Figure 4) suggest the lack of phylogenetic structure within the complex. Excluding *Calligonum arich* (5 individuals), *Calligonum ebinuricum* (3 individuals) can be easily differentiated from the *Calligonum mongolicum* complex (13 individuals) (Figures 3 and 4). The intermixed patterns of sequences from different “species” of the *Calligonum mongolicum* complex may indicate past or present introgressive potential of the *Calligonum mongolicum* complex and argues for the existence of hybridisation or interbreeding (if these “species” represent the same taxon).

### Implications on taxonomy and conservation of *Calligonum*

*Calligonum* is one of the medium-sized genera of Polygonaceae with approximately 60–80 species and represents a rapid diversification in the hot and arid deserts of Central Asia to western China (Mabberley 1990). Molecular analyses of both nrDNA ITS and some cpDNA sequences (*trn*L-F, *mat*K, *atp*B-*rbc*L, *psb*A-*trn*H, *psb*K-*psb*L and *rbc*L) have not resolved relationships amongst species of *Calligonum* (Sanchez et al. 2011, Sun and Zhang 2012, Li et al. 2014). Our study showed that *Calligonum ebinuricum* possesses highly distinct nrITS sequences (Figures 3 & 4); yet the ITS and ETS sequences of the *Calligonum mongolicum* complex generated a topology with the species of the complex highly intermixed with each other in the tree. The authors’ results both in this paper and in their previous studies (Shi et al. 2011, 2012, 2013, 2016, Shi and Pan 2015) argue for the merging of *Calligonum chinense*, *Calligonum gobicum*, *Calligonum pumilum* and *Calligonum zaidamense* with *Calligonum mongolicum* as proposed by Soskov (1975a, 1975b). Detailed evidence was also recently presented on merging *Calligonum pumilum* with the more widespread *Calligonum mongolicum* (Shi et al. 2016). Detailed morphological comparisons of the other species in the complex will be pursued by the authors as was done for *Calligonum pumilum* and *Calligonum mongolicum* (Shi et al. 2016) and the phylogeographic structure of the complex will be further explored with phylogenomic methods (Wen et al. 2015, Zimmer and Wen 2015).

Distributional ranges of some species in clade C (Figure 3) do not overlap but are geographically close or adjacent to each other. *Calligonum roborowskii* (2*n*=36) grows at the edge of Taklamakan basin; *Calligonum taklamakan* occurs in the central part of the basin; and the other species in the complex except *Calligonum mongolicum* are confined to the south-eastern edge of the basin and *Calligonum ebinuricum* is in North Xinjiang and also in Mongolia but never in South Xinjiang. According to their morphological comparisons (Gulinuer 2008, Kang et al. 2008), the taxonomic relationship of *Calligonum ebinuricum* and *Calligonum taklamakan* with other species needs further analyses. The fact that most of the collected seeds can germinate without any pre-treatment suggests that the five *Calligonum* species produce enough seeds to renew the populations. On the other hand, the *ex-situ* conservation of genetic diversity for the long-term survival of species of *Calligonum* needs a new management strategy due to their reproductive biology and the potential for hybridisation/interbreeding (Kramer and Havens 2009, Swarts and Dixon 2009). Special efforts are needed to ensure isolation of genetic sources in *ex situ* conditions.
